# MDMA modulates human sensorimotor cortical pathways during gentle touch

**DOI:** 10.1162/imag_a_00316

**Published:** 2024-10-17

**Authors:** Hanna Molla, Giovanni Novembre, Anya Bershad, Linda Handlin, Irene Perini, Harriet de Wit, India Morrison

**Affiliations:** Department of Psychiatry and Behavioral Neuroscience, University of Chicago, IL, United States; Department of Biomedical and Clinical Sciences, Cell and Neurobiology Division, Linköping University, Linköping, Sweden; Department of Psychiatry, University of California Los Angeles, Los Angeles, CA, United States; Department of Biomedicine, School of Health Sciences, University of Skövde, Skövde, Sweden; Center for Social and Affective Neuroscience, Department of Biomedical and Clinical Sciences, Linköping University, Sweden; Center for Medical Image Science and Visualization (CMIV), Linköping University, Linköping, Sweden

**Keywords:** MDMA, affective touch, fMRI, social, oxytocin, social touch

## Abstract

The stimulant ± 3,4-methylenedioxymethamphetamine (MDMA) has been shown to enhance the perceived pleasantness of touch. However, the underlying neural processes contributing to touch-related effects of MDMA are not well understood. Using a double-blind, randomized, within-subject design, this study used fMRI to examine hemodynamic changes following MDMA (1.5 mg/kg) vs. lactose placebo administration during gentle touch stimulation in a healthy sample (*N*= 18). Participants were stroked on the forearm at a slower, more pleasant (3 cm/s), and a faster (30 cm/s), less pleasant speed. For the MDMA session, participants’ affective ratings of touch stimulation were higher than their placebo ratings. Increase in plasma oxytocin (OT) levels was also greater during the MDMA session. On the neural level, primary sensorimotor areas showed greater hemodynamic changes during the MDMA than during the placebo session for both touch speeds, indicating a relatively early influence within somatosensory pathways. Changes in OT levels showed an interaction with drug in an occipitotemporal region, area MT+, associated with motion perception. However, posterior insula did not show preferential activation for the slower stroking speed. These initial findings provide a basis for extending our knowledge of the neural processes underlying the effect of MDMA on affective touch.

## 1 Introduction

Recreational users of the methamphetamine derivative 3,4-methylenedioxy-methamphetamine (MDMA) report that the drug heightens perception of touch and increases desire for physical contact (Cole & Sumnall, 2003). This is consistent with its “empathogenic” acute effects, which include subjective reports of social connectedness, sociability, interpersonal closeness, and overall well-being (Bedi et al., 2010;Holze et al., 2020;Hysek et al., 2014;Kirkpatrick et al., 2014;Molla et al., 2023). Such effects are also thought to contribute to its efficacy in therapeutic settings as an adjunct to psychotherapy, for treating disorders such as PTSD. However, the pharmacological effects of the drug are complex and the underlying neural mechanisms of such effects remain unclear. Considering the centrality of affective touch perception in the experience of MDMA users and in therapeutic contexts, examining the effects of the drug on affective touch provides a route to insight into the underlying pharmacological and neural mechanisms.

In general, slower speeds of gentle skin stroking (around 3 cm/s) are rated as more pleasant than faster ones (around 30 cm/s) (Löken et al., 2009;Morrison et al., 2010;Sailer et al., 2020), and in one study, MDMA enhanced this difference (Bershad et al., 2019). At the neural level, both slower stroking speeds and positively evaluated touch in general preferentially modulate hemodynamic activation in the posterior insular and inferior parietal regions (Morrison, 2016;Morrison et al., 2011;Perini et al., 2015). Investigating such differential processes involved in affective touch can provide insight on how MDMA affects cortical networks underlying the evaluation of affective stimuli more broadly.

For example, one possibility is that MDMA affects touch perception by modulating posterior insula (PI) and/or other regions previously associated with affective touch, such as superior temporal cortex (Davidovic et al., 2016;Gordon et al., 2013;Kaiser et al., 2016). In this case, greater blood-oxygen-level-dependent (BOLD) activation changes for MDMA would be expected in these regions, as compared with placebo. Another possibility is that MDMA acts by modulating primary somatosensory pathways. It is also possible that MDMA modulates integrative processing of touch information across broader cortical networks beyond these key tactile and affective processing pathways.

MDMA increases extracellular levels of serotonin, and to a lesser extent, dopamine and norepinephrine (Simmler & Liechti, 2018), as well as the neuropeptide hormone oxytocin (OT) (Holze et al., 2020;Kirkpatrick et al., 2014). Thus, any effects of MDMA on neural processing of sensory and affective information may be related to OT changes. Both OT and touch are key mediators of social attachment across many mammalian species (Bos et al., 2012;Tang et al., 2020), and social tactile stimulation such as affectionate stroking has elicited endogenous OT release in adult humans (Handlin et al., 2023). This touch-evoked increase in OT has recently been tied to changes in BOLD activity in temporal and parietal regions involved in integrating sensory and contextual information in social situations (Handlin et al., 2023), raising the possibility that OT is linked to the effects of MDMA on neural processing of social touch.

We, therefore, investigated the influence of MDMA on blood-oxygen-level-dependent (BOLD) signal and plasma OT levels on touch processing during slower and faster stroking speeds. In a within-subject, randomized design, participants received MDMA (1.5 mg/kg) on one visit and placebo on another, and experienced slow (3 cm/s) and fast (30 cm/s) brush stroking on their forearm during an fMRI scan on each visit. Blood samples for OT analysis were obtained before and after drug administration. During each session, participants completed questionnaires related to drug effects and affective experience. We expected that MDMA would increase perceived touch pleasantness and plasma OT levels, as well as altering processing in insular, parietal, and temporal cortex regions associated with affective touch and socially relevant stimuli (Allison et al., 2000;Igelstrom & Graziano, 2017). Based on previous research (Handlin et al., 2023), we also hypothesized that MDMA would alter covariation between OT and BOLD in parietotemporal pathways.

## 2 Methods

### 2.1 Participants

The study was approved by the Institutional Review Board of the Biological Sciences Division of the University of Chicago. It was registered on Clinicaltrials.gov as NCT04053036 (“Effects of Drugs on Responses to Brain and Emotional Processes”).

Twenty-two healthy volunteers (13 male, 9 female, age 21–40 years, mean age 27.8 ± 2.9) participated in the study at University of Chicago (Table 1). Screening included a physical examination, electrocardiogram (EKG), psychiatric and medical history, and drug use history assessment. Inclusion criteria were previous lifetime drug use (reported MDMA use), BMI 19–30, fluency in English, and minimum education level of a high school diploma (in the United States, completed about age 17–18 years). Exclusion criteria were high blood pressure, abnormal EKG, severe lactose intolerance, any medical condition requiring regular medication, current DSM-V diagnosis of a mood, anxiety, or psychotic disorder, history of dependence on stimulant drugs, claustrophobia, metal implants, pregnancy, and use <25 cigarettes per week to mitigate possible nicotine withdrawal effects during study sessions.

**Table 1.** Demographics.

Participant demographics	
	N or mean (% or SD)
Sex	
Male	13 (59.1 %)
Female	9 (40.9%)
Race	
Asian	3 (13.6%)
Black	0 (0.0%)
Caucasian	15 (68.2%)
Other/more than one	4 (18.2%)
Age	27.8 (2.9)
BMI	24.2 (2.6)
Current drug use	
Caffeinated drinks per day	1.20 (0.57)
Cigarettes per day	0.46 (1.61)
Alcoholic drinks per week	1.82 (1.99)
Lifetime drug use (at least once)	
Cannabis	21 (95.5%)
Sedatives	7 (31.8%)
Stimulants (other than MDMA)	15 (68.2%)
Opiates	7 (31.8%)
Psychedelics	21 (95.5%)
Median lifetime use of MDMA	10
Range of lifetime use of MDMA	3 – 40 *
Median last use of MDMA (in years)	0.67
Range of last use of MDMA (in years)	0.005 – 8.0

* Note: one subject reported lifetime use of MDMA to be greater than 40 with last use being 8 years prior to the start of the study.

The sample size was limited by availability of participants meeting all inclusion criteria during the COVID-19 pandemic. Power calculations were based on a previous study (Bershad et al., 2019) in which the effect size for mean differences between 3 cm/s stroking following MDMA administration compared with placebo was 0.52 (Cohen’s d). Post hoc power analyses in our current study indicates that achieved power was 0.66, α = 0.05 (two-tailed; G*Power 3.1.9.7). (A sample size of n = 30 would be needed for power = 0.80, α = 0.05, two-tailed).

### 2.2 Procedure

#### 2.2.1 Orientation

During an initial orientation session, participants were informed about the study procedures and provided written consent. To minimize drug expectancies, participants were told they might receive a placebo, stimulant including amphetamine or MDMA, a sedative/tranquilizer such as Valium, or an opiate drug. They were instructed to abstain from drug use for at least 2 days and alcohol use for 24 hours before the sessions.

#### 2.2.2 Study sessions

The two 4.5-hour sessions were separated by at least 4 days. Upon arrival in the laboratory at 9 am, participants provided urine (CLIAwaived Instant Drug Test Cup) and breath (Alco-Sensor III, Intoximeters, St Louis, MO) samples to screen for recent drug and alcohol use, and pregnancy for women. Participants who tested positive for any substances were rescheduled. Participants then completed predrug measures of subjective mood, cardiovascular, and drug effects, and provided a plasma sample for OT analysis. At 10 am, subjects ingested a capsule containing 1.5 mg/kg MDMA (Organix Inc, MA; opaque size 00 capsules with lactose [USP] filler) or placebo (lactose only) and relaxed in a comfortable room to allow for drug absorption. The drug was administered in randomized order under double-blind conditions. At 11:30 am, subjects were placed in the scanner, where they underwent a touch stimulation paradigm (see description below andFig. 1A). At regular intervals (30, 60, 180, 210 min postdrug), cardiovascular measures (blood pressure and heart rate) were obtained and all questionnaires were administered. A second plasma sample was collected at 12:30 pm. Participants were discharged at 1:30 pm.

**Fig. 1.**
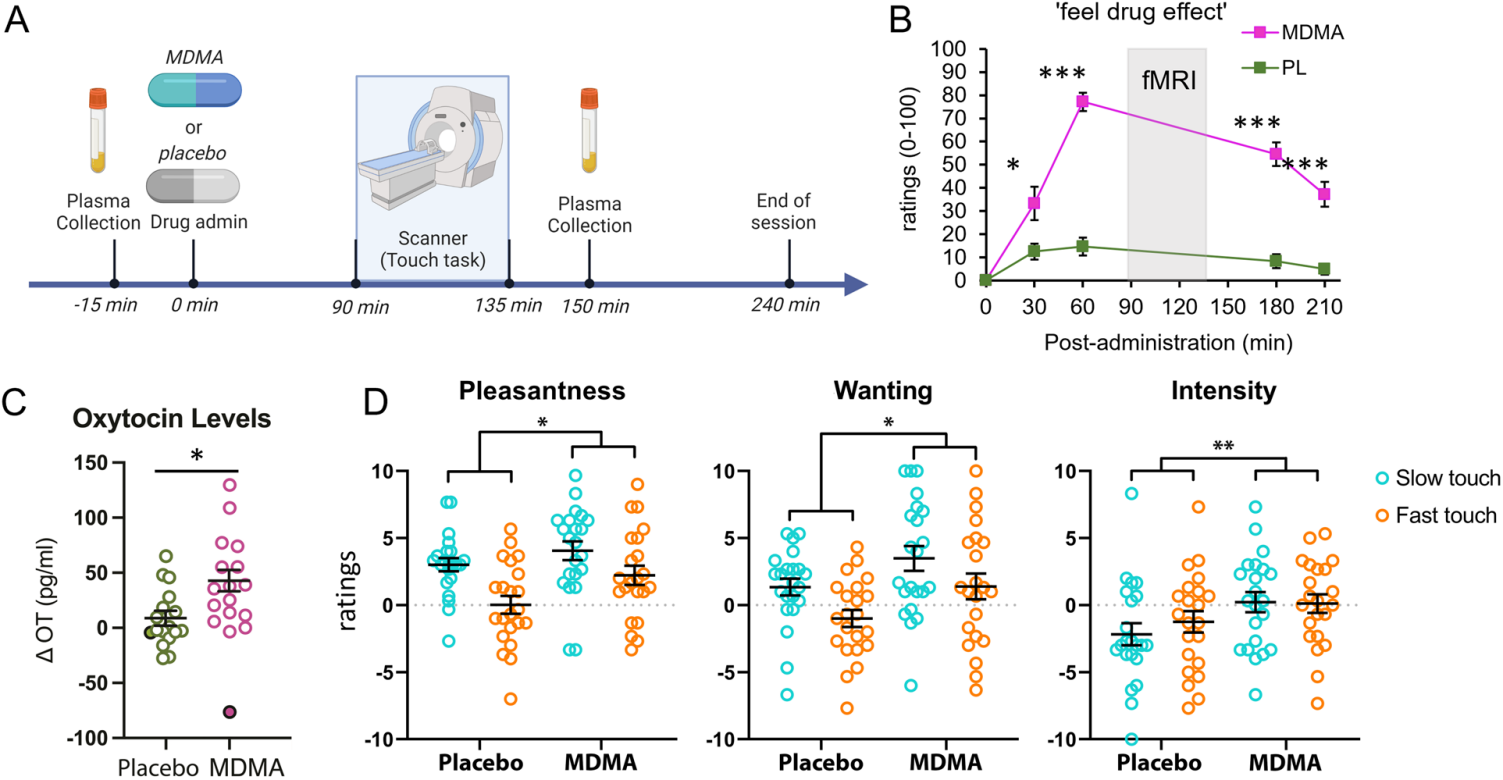
Experiment timeline and subjective effects. (A) Session timeline with drug administration and plasma sample collection. (B) Effects of MDMA on ratings of feeling a drug effect. MDMA vs. placebo, ***p < 0.005. (C) Mean (± SEM ) change in plasma oxytocin (post- minus precapsule administration) for placebo (green) and MDMA (purple) conditions. *p< 0.05. Filled dots represent the corresponding values for the excluded outlier with (>3 SD). (D) Mean (± SEM) ratings of pleasantness, wanting more, and intensity for slower (3 cm/s) touch (turquoise) and faster (30 cm/s) touch (orange). Main effect of drug,*p< 0.05 (pleasantness and intensity), **p< 0.01 (wanting).

#### 2.2.3 Questionnaires

The Profile of Mood States—(POMS) (McNair et al., 1971), which consists of 72 adjectives commonly used to describe momentary mood states, was completed at baseline and at intervals throughout the session (seeFig. 1A). Drug Effects Questionnaire—(DEQ) (Fischman & Foltin, 1991) consists of questions on a visual analog scale (VAS) about the subjective effects of drugs. Participants were asked to rate on a 100 mm line the extent to which they felt a drug effect, whether they liked or disliked the drug effect, and would they have wanted to take more of the drug if given a choice. Subjects completed VAS with adjectives that describe MDMA effects, including “Insightful,” “Sociable,” “Confident,” “Lonely,” “Playful,” “Dizzy,” “Loving,” “Friendly,” and “Restless.” All measures were completed pre-, +30, +60, +180, and +210 min postdrug administration.

### 2.3 Affective touch

Gentle touch stimulation was administered while participants were in the scanner. Participants were gently stroked by a research assistant, in a back and forth, proximal to distal motion on the left forearm using a hand-held 5 cm wide goat hair brush applied to a 9 cm section of the forearm (Ackerley, Carlsson, et al., 2014;Triscoli et al., 2013). A brush was used instead of hand-to-arm contact to minimize associated variables such as changes in skin temperature (Ackerley, Backlund Wasling, et al., 2014). The experimenter wore headphones to receive metronomic audio cues to pace brushing velocity. Stimuli were presented with Presentation software. The paradigm was a 2 x 2 factorial design, with factors drug (MDMA or placebo, within-subject by session) and stroking velocity (3 cm/s or “slow” and 30 cm/s or “fast”). The tactile stimulation consisted of 3 runs of 10 12s trials each, 5 at each of the 2 stroking speeds (15 trials/condition total), with the presentation of velocity pseudo-randomized and counterbalanced within and between each block. Participants were instructed to maintain gaze at a fixation cross on the screen during the touch trials to minimize eye movement. Each trial was followed by a 6s stimulus rating task in 6 of the 10 touch trials per run. The intertrial interval was jittered at 7s, 9s, 10s, or 12s, counterbalanced so that each run had the same duration. For the six post-trial ratings in each run, participants were asked to rate (once per speed per run): “How pleasant does the stimulation feel?” (anchors: unpleasant; pleasant); “How intense does the stimulation feel?” (anchors: not intense; intense); and “How much would you like to receive the stimulation again?” (anchors: not at all; very much), using a visual analog scale from -10 to +10. Rating scores for each condition were derived from individuals’ means over the three runs for each rating type.

### 2.4 Neuroimaging

#### 2.4.1 fMRI data acquisition

Imaging was performed using a Phillips Achieva dStream 3T MRI scanner. Three gradient echoplanar imaging (EPI) scans were acquired to measure responses to the tasks (168 volumes; TR = 2000 ms; echo time = 28 ms; flip angle = 77°; 39 3 mm-thick axial slices; slice gap 0.5 mm; acquisition matrix = 64 × 62; voxel size = 3.0 × 3.0 × 3.0 mm^3^; SENSE factor = 2). Structural scans were also acquired using a T1-weighted magnetization-prepared rapid gradient echo (MPRAGE) sequence (TR/TE = 8/3.5 ms; TI = 960 ms; flip angle = 8 degrees; SENSE factor = 2; FOV 240 x 228 mm; matrix size = 240 x 228; 171 sagittal slices; voxel size = 1 x 1 x 1 mm^3^) for coregistration and normalization to the Montreal Neurological Institute (MNI) coordinate system. Head movement was minimized by using a conformal pillow which is fitted to the participant’s head. The scan took 45 min to complete.

#### 2.4.2 fMRI preprocessing and whole brain analysis

Preprocessing and statistical analysis of MRI data were performed using Analysis of Functional Neuroimages (AFNI) statistical software (version 19.1.12 (Cox, 1996)). Functional data were first despiked. Each EPI volume and the T1 were then aligned to the EPI volume with the minimum outlier fraction (using the AFNI outlier definition) to correct for motion. Functional images were warped to the MNI 152 template using a combination of affine and nonlinear transformations (Talairach & Tournoux, 1988). Finally, spatial smoothing was applied with a 4 mm full-width at half-maximum filter.

Residual effects of head motion were corrected by including the estimated motion parameters (and their first-order derivatives) as regressors of no interest. A motion censoring threshold of 0.4 mm per TR was implemented in combination with an outlier fraction threshold of 0.05. Volumes violating either of these thresholds were subsequently ignored in the time series regression. This led to the exclusion of four participants, whose MDMA sessions had 28% or more censored volumes at least in one condition (slow or fast touch). fMRI data analyses were, therefore, performed only on data from the remaining 18 participants. Generally, more motion occurred during MDMA sessions compared with placebo sessions. To quantify this further, we extracted a motion parameter invariant to censoring thresholds—that is, average motion per TR—and used this value for each participant and each session as a covariate of no interest in the analysis (see below). A paired*t*-test on this parameter confirmed that average motion during MDMA sessions (Mean ± SEM = 0.129 ± 0.015) was significantly higher than during placebo sessions (Mean ± SEM = 0.078 ± 0.038;*t*_17_= 4.706,*p*< 0.001).

For each remaining participant (N = 18), whole-brain voxel-wise general linear models (GLM) were created for each of the two sessions using 3dDeconvolve. One regressor (convolved with a standard model of the hemodynamic response function, HRF) modeled the final 12 s of each of the touch conditions, discarding the first 3 s to exclude any delay and onset-related activity during the beginning of the trial. We also included additional regressors of no interest to model the effects of motor responses during the rating of the touch stimuli.

At the group level, statistical maps were first set at a per-voxel*p*value threshold of 0.002, and then the AFNI program 3dClustSim was used to determine cluster-size thresholds for identifying effects significant at α = 0.05 family-wise-error (FWE) corrected within cluster (Cox et al., 2017). Average spatial smoothness estimates, across all participants, used by 3dClustSim were obtained using the 3dFWHMx function with the ACF flag, as per current AFNI recommendations (Cox et al., 2017) to achieve multiple comparison corrected results without inflated false positives (Eklund et al., 2016).

#### 2.4.3 Linear mixed-effects modeling with OT and pleasantness rating covariates

To examine BOLD activation related to Drug Treatment (MDMA, Placebo) and Touch Velocity (slow touch, fast touch), and their respective covariation and interaction with OT levels and pleasantness ratings, we performed a linear mixed-effects modeling analysis (3dLMEr in AFNI (Chen et al., 2013)). The model included for each participant four statistical maps from the single-level analysis following the 2 x 2 factorial design (Drug Treatment* Touch Velocity), plus covariates of interest (OT and pleasantness) and of no interest (Movement). Random effects were OT and pleasantness slopes across subjects, with correlated intercept. For OT, prepost capsule differences were entered as covariate (one value per Drug Treatment). For pleasantness ratings, average values for each condition were entered as covariate (one value for each of the four conditions). Finally, the significant different amount of motion between drug treatments was accounted for by the average motion per TR. Before entering the model, OT and average motion per TR values were centered around the mean of each drug treatment, whereas the pleasantness ratings were centered around the mean of each respective condition. Five of the 18 participants included in the MRI data analysis lacked OT values for at least one time point: for two of them both pre–post differences could not be calculated, whereas for the remaining three, only one Δ value could not be calculated. For these participants, the mean value of the respective drug treatment was considered as OT covariate (which corresponded to zero after mean centering).

### 2.5 Cardiovascular measures

Blood pressure and heart rate were monitored using portable blood pressure cuffs (Omron BP791IT, Omron Healthcare).

### 2.6 Hormonal measures

Plasma samples for OT were collected before and 120 min after drug administration. Plasma samples for OT analysis were extracted using acetonitrile precipitation (Merck Millipore: Human Neuropeptide Magnetic Bead Panel 96-Well Plate Assay Cat. # HNPMAG-35K), and OT concentrations were then determined using the Oxytocin ELISA kit (Enzo Life Sciences; sensitivity > 15.0 pg/ml, intra-assay precision 10.2–13.3% CV, interassay precision 11.8–20.9% CV). Standards and controls were implemented according to manufacturer recommendations. Washing procedures were performed using a Wellwash Microplate Washer (ThermoFisher Scientific, Waltham, Massachusetts, USA) and the absorbance was read using a Multiskan FC Microplate Photometer (ThermoFisher Scientific, Waltham, Massachusetts, USA). The color development of the samples was read for OT at 405 nm (background correction at 571 nm). SkanIt Software was used for the creation of standard curves, curve fitting, and calculation of concentrations (ThermoFisher Scientific, Waltham, Massachusetts, USA). Plasma samples were not obtained for five of the participants.

### 2.7 Data analysis

The subjective and cardiovascular measures were analyzed by calculating the peak change from baseline (predrug) on each session. Two-tailed, paired samples*t*-tests (SPSS Version 25) were used to compare peak change subjective and cardiovascular measures during MDMA vs. placebo sessions. On the DEQ “feel drug” item, a repeated measures ANOVA was conducted to examine time course specific drug effects using mean ratings at each time point from drug and placebo sessions.

A secondary research question in the present study was whether MDMA would increase plasma OT, as reported previously (Kirkpatrick et al., 2014). For the present study, the achieved power to detect this effect was 0.68, Cohen’s d, α = 0.05, two-tailed. (A sample size of n = 19 would be needed for power = 0.80, α = 0.05, two-tailed). Changes in OT pre- and postcapsule administration were compared for MDMA and placebo sessions using a two-tailed, paired samples*t*-test.

## 3 Results

### 3.1 Demographics

Most participants were in their late 20’s with moderate prior drug use (Table 1). Median lifetime use of MDMA was 10 times.

### 3.2 MDMA effects on subjective drug, mood, and physiological measures

MDMA increased ratings of “feeling a drug effect” compared with placebo (drug x time point,*F*_1,21_= 26.43,*p < *0.001, at 30, 60, 180, and 210 min;Fig. 1B). MDMA elicited the expected subjective and cardiovascular effects, similar to what has been reported previously (Table 2) (Bershad et al., 2016).

**Table 2.** Subjective and cardiovascular measures (peak change from baseline values).

Measure	PLC (SD)	MDMA (SD)	*t(21), p*
DEQ (0-100)			
Feel drug effect	19.7 (18.9)	81.2 (16.8)	10.3, 0.000***
Like drug	34.4 (29.5)	85.0 (16.3)	8.4, 0.000***
Dislike drug	18.5 (23.9)	42.3 (23.7)	3.2, 0.004**
High	19.5 (23.1)	79.7 (15.5)	11.2, 0.000***
Want more	39.0 (34.0)	73.8 (27.6)	4.7, 0.000***
VAS (0-100)			
Elated	2.5 (26.2)	42.7 (33.1)	4.8, 0.000***
Insightful	-3.5 (18.5)	33.0 (33.6)	4.2, 0.000***
Sociable	-1.3 (25.4)	25.1 (35.5)	2.9, 0.008*
Confident	-3.9 (21.7)	14.0 (29.9)	2.1, 0.04*
Playful	-4.2 (27.2)	28.3 (39.8)	3.1, 0.005*
Loving	0.2 (25.3)	28.9 (41.2)	2.3, 0.03*
Friendly	-6.5 (24.5)	26.8 (26.8)	4.6, 0.000***
Restless	-3.3 (26.2)	32.6 (32.5)	4.0, 0.000***
Stimulated	-7.6 (25.4)	38.5 (44.6)	4.8, 0.000***
Sedated	18.2 (36.2)	27.8 (40.4)	0.8, 0.4
Anxious	-0.6 (20.0)	17.2 (24.3)	3.5, 0.002**
Lonely	4.5 (19.0)	11.1 (32.5)	0.8, 0.4
Dizzy	12.8 (21.7)	37.0 (27.3)	3.3, 0.004**
POMS			
Friendliness	-3.7 (3.9)	2.6 (7.5)	3.9, 0.000***
Elation	-0.8 (4.6)	4.7 (6.6)	3.0, 0.007*
Vigor	-4.0 (5.6)	3.3 (9.9)	3.0, 0.007*
Fatigue	0.7 (4.5)	3.2 (6.6)	1.7, 0.1
Anger	-0.8 (2.4)	1.0 (5.9)	1.4, 0.2
Anxiety	-1.9 (4.2)	4.2 (7.8)	3.8, 0.001**
Depression	-0.5 (4.2)	3.5 (7.9)	2.0, 0.06
Confusion	0.2 (2.9)	3.7 (6.4)	2.5, 0.02*
Physiological			
Systolic BP	-2.6 (18.2)	26.8 (21.7)	4.3, 0.000***
Diastolic BP	-5.2 (17.8)	17.5 (13.4)	5.0, 0.000***
HR	3.2 (15.0)	26.9 (29.4)	3.4, 0.003**

*p≤ 0.05, **p≤ 0.01, ***p≤ 0.001.

MDMA increased OT levels (postdrug—predrug) compared with placebo (*p*= 0.02; one outlier with values of >3 SD of the mean in the MDMA condition was removed;Fig. 1C). There was no relationship between drug-induced change in OT and ratings of touch pleasantness.

### 3.3 MDMA effects on affective touch

Ratings of touch pleasantness generally increased during the MDMA session, relative to placebo (main effect of drug,*F*_1,21_= 5.92,*p = *0.02) and were higher for the slower relative to the faster stroking speed (main effect of touch velocity,*F*_1,21_= 16.11,*p = *0.001).

Ratings of touch “intensity” were greater for MDMA than for placebo (main effect of drug,*F*_1,21_= 7.58,*p = *0.01), regardless of stroking velocity. Ratings for “wanting more touch” were greater for the slower speed (main effect of touch velocity,*F*_1,21_= 11.37,*p = *0.003) and for MDMA than for placebo (main effect of drug,*F*_1,21_= 8.92,*p = *0.007), but there were no interactions with speed of touch (Fig. 1D). In MDMA sessions, pleasantness ratings were not significantly correlated with plasma OT regardless of speed (r = 0.02,*p = *0.9 for the faster speed; r = 0.07,*p = *0.8 for the slower speed; outlier data as excluded).

### 3.4 fMRI

The linear mixed-effects modeling analysis included the factors Drug (MDMA, placebo) and Touch Velocity (slower, faster), with covariates of interest (OT and pleasantness) and no interest (movement). This model did not reveal above-threshold interaction between Drug (MDMA, placebo) and Touch Velocity (slower, faster). Main effects of Drug and Velocity were observed, as well as interactions with covariates, described below. BOLD activations from the linear mixed-effects modeling are summarized inTable 3.

**Table 3.** Linear mixed-effects modeling with factors drug treatment (placebo, MDMA), brushing velocity (slow, fast), with OT changes and mean pleasantness ratings as covariates.

Main effect: drug treatment		
MDMA > placebo		
Cluster (size)	Peaks locations	*Chi-sq* (x, y, z)
#1 (168)	Right precentral gyrus	36.06 (46, -11, 55)
#2 (118)	Left calcarine gyrus	22.62 (-23, -65, 4)
#3 (88)	Left postcentral gyrus	30.02 (-23, -29, 61)
#4 (35)	Left postcentral gyrus	21.12 (-47, -17, 52)
#5 (30)	Left middle frontal gyrus	29.76 (-32, 40, 34)
#6 (24)	Right middle temporal gyrus	21.61 (55, -62, 16)
#7 (19)	Right supramarginal gyrus	25.67 (64, -35, 25)
#8 (19)	Left middle cingulate cortex	23.62 (-8, -5, 46)
#9 (17)	Right calcarine gyrus	26.28 (28, -62, 4)
#10 (15)	Right mid orbital gyrus	18.66 (1, 52, -11)
#11 (15)	Left middle frontal gyrus	24.98 (-23, 4, 52)
#12 (12)	Left inferior temporal gyrus	26.98 (-59, -56, -14)
#13 (12)	Right mid orbital gyrus	33.11 (4, 40, -14)
#14 (12)	Right calcarine gyrus	25.48 (19, -80, 7)
#15 (12)	Right superior frontal gyrus	25.02 (19, -8, 73)
Placebo > MDMA		
#1 (43)	Right middle occipital gyrus	23.71 (40, -65, 7)
#2 (28)	Right superior occipital gyrus	23.63 (28, -80, 25)
#3 (25)	Right cerebellum	27.21 (34, -56,-44)
#4 (25)	Left middle occipital gyrus	24.44 (-29, -98, 10)
#5 (18)	Left middle occipital gyrus	18.38 (-29, -86, 22)
#6 (17)	Right superior parietal lobule	21.24 (28, -59, 55)
#7 (16)	Left cerebellum	30.86 (-23, -68, -47)
#8 (16)	Left inferior occipital gyrus	18.68 (-23, 89, -8)
#9 (15)	Left inferior occipital gyrus	18.71 (-47, -77, -5)
#10 (14)	Left cerebellum	25.26 (-32, -71, -56)
#11 (14)	Right cerebellum	21.28 (10, -71, -44)
#12 (12)	Left cerebellum	25.75 (-23, -44, -53)
#13 (12)	Right inferior temporal gyrus	22.10 (49, -62, -5)
#14 (12)	Right middle occipital gyrus	22.68 (31, -89, 13)
#15 (12)	Left middle occipital gyrus	21.39 (-29, -83, 34)
Main effect: brushing velocity		
Cluster (size)	Peaks locations	*Chi-sq* (x, y, z)
Fast > slow		
#1 (50)	Right postcentral gyrus	29.00 (34, -29, 64)
#2 (15)	Right middle temporal gyrus	20.71 (52, -65, -2)
#3 (12)	Right middle occipital gyrus	18.71 (37, -86, 7)
Slow > fast		
#1 (25)	Left inferior parietal lobule	16.61 (-41, -38, 49)
Main effect: pleasantness		
Cluster (size)	Peaks locations	*Chi-sq* (x, y, z)
#1 (19)	Left supplementary motor area	22.33 (-2, -5, 52)
#2 (17)	Right supplementary motor area	20.29 (4, -26, 61)
#3 (12)	Left insular lobe	22.32 (-35, -11, 13)
Interaction: drug treatment * *ΔOT*		
Cluster (size)	Peaks locations	*F* (x, y, z)
#1 (46)	Left calcarine gyrus	24.12 (1, -77, 16)
#2 (43)	Left inferior temporal gyrus	29.89 (-59, -56, -14)
#3 (43)	Left calcarine gyrus	26.71 (-11, -104, -2)
#4 (21)	Right middle occipital gyrus	20.43 (34, -89, 1)
#5 (17)	Right calcarine gyrus	23.19 (10, -92, -2)
#6 (15)	Left calcarine gyrus	20.00 (-20, -62, 4)
#7 (14)	Left middle frontal gyrus	32.16 (-32, 19, 55)
#8 (13)	Left middle temporal gyrus	20.23 (-47, -62, 1)
#9 (12)	Left cerebellum	20.92 (-17, -80, -50)
#10 (12)	Right inferior temporal gyrus	19.62 (40, -65, -5)
#11 (12)	Right precentral gyrus	28.80 (19, -29, 70)
Interaction: drug treatment*pleasantness		
Cluster (size)	Peaks locations	*F* (x, y, z)
#1 (85)	Left lingual gyrus	26.24 (-20, -65, -8)
#2 (49)	Right supplementary motor area	26.75 (4, -5, 61)
#3 (30)	Left superior temporal gyrus	30.84 (-62, -35, 13)
#4 (30)	Left paracentral lobule	26.80 (-2, -35, 64)
#5 (22)	Left calcarine gyrus	18.26 (-17, -71, 13)
#6 (14)	Left calcarine gyrus	23.78 (-17, -56, 74)
#7 (12)	Left superior temporal gyrus	27.98 (-47, -17, 4)

All contrasts thresholded at*p < *0.002, cluster-size thresholded at alpha = 0.05 FWE for n = 18 functional datasets. For each cluster under each contrast heading, size in voxels, location, maximum*chi-square*score, and MNI coordinates (x, y, z) are given.

To explore the achieved effect size for the main clusters of interest, we performed two*t*-tests, one for each main effect (Drug and Touch Velocity). We then matched the original clusters in each main effect to those found in the corresponding*t*-test to estimate the peak effect size, considering there is no standard procedure for obtaining these values for a chi-square statistic in AFNI. These values are reported below for each main effect.

### 3.5 Effect of MDMA on BOLD activation

**Fig. 2.**
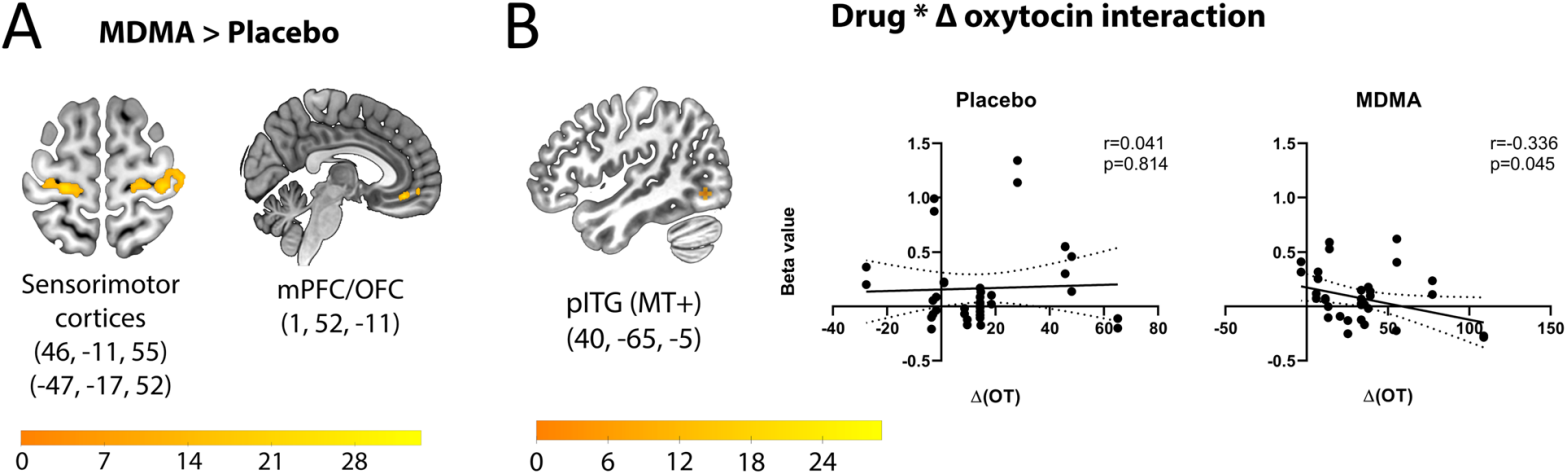
BOLD modulation by drug and change in plasma oxytocin. (A) Main effects of drug (MDMA > placebo) in sensorimotor, medial prefrontal and orbitofrontal cortices (mPFC/OFC). See alsoTable 2. (B) BOLD activation in the posterior inferior temporal gyrus (pITG/MT+) showed an interaction between drug and changes in plasma oxytocin (post- minus predrug levels), with a more negative relationship between BOLD and oxytocin change during the MDMA session. Images shown in radiological convention (left side of image is right side of brain). Color bars represent*F*statistic values.

MDMA, regardless of touch velocity, elicited greater activation across 15 clusters, relative to placebo (Table 3). Regions that showed greater activation during touch after MDMA included the right precentral gyrus (peak effect size = 0.294), left and right calcarine gyrus (two clusters), left postcentral gyrus (two clusters; peak effect sizes = 0.200 and 0.197), left middle frontal gyrus (two clusters), right middle temporal gyrus, right supramarginal gyrus (peak effect size = 0.157), left middle cingulate cortex, right mid orbital gyrus (two clusters), left inferior temporal gyrus, and right superior frontal gyrus (Fig. 2A). Relative to placebo, MDMA also decreased activation in the right and left middle occipital gyrus (two clusters each; left peak effect sizes = 0.349 and 0.300), right superior occipital gyrus (peak effect size = 0.236), left and right cerebellum (three and two clusters each, respectively; left peak effect size = 0.225; right peak effect size = 0.693), right superior parietal lobule, left inferior occipital gyrus (two clusters; peak effect size = 0.213), and right inferior temporal gyrus.

### 3.6 Effect of touch velocity on BOLD activation

Regardless of drug administration (MDMA or placebo), slow touch elicited higher activity in the left inferior parietal lobule (peak effect size = 0.155), relative to fast touch. Fast touch elicited higher activation in the right postcentral gyrus (peak effect size = 0.227), the middle temporal gyrus, and the right middle occipital gyrus (peak effect size = 0.187), relative to slow touch.

### 3.7 Relationship between BOLD activation and OT

A secondary hypothesis of this study was that the MDMA-mediated increase in OT would covary with BOLD changes in parietotemporal pathways formerly implicated in touch processing. BOLD activation correlated more positively with plasma OT changes (post- minus predrug) after MDMA in seven clusters: the left and right calcarine gyrus (three and one clusters, respectively), the left inferior frontal gyrus, the left middle frontal gyrus, and the right precentral gyrus. Four clusters showed a more positive relationship under placebo rather than MDMA: the right middle occipital gyrus, the left middle temporal gyrus, the left cerebellum, and the right inferior temporal gyrus (MT+;Fig. 2B).

### 3.8 Relationship between pleasantness ratings and BOLD activation

**Fig. 3.**
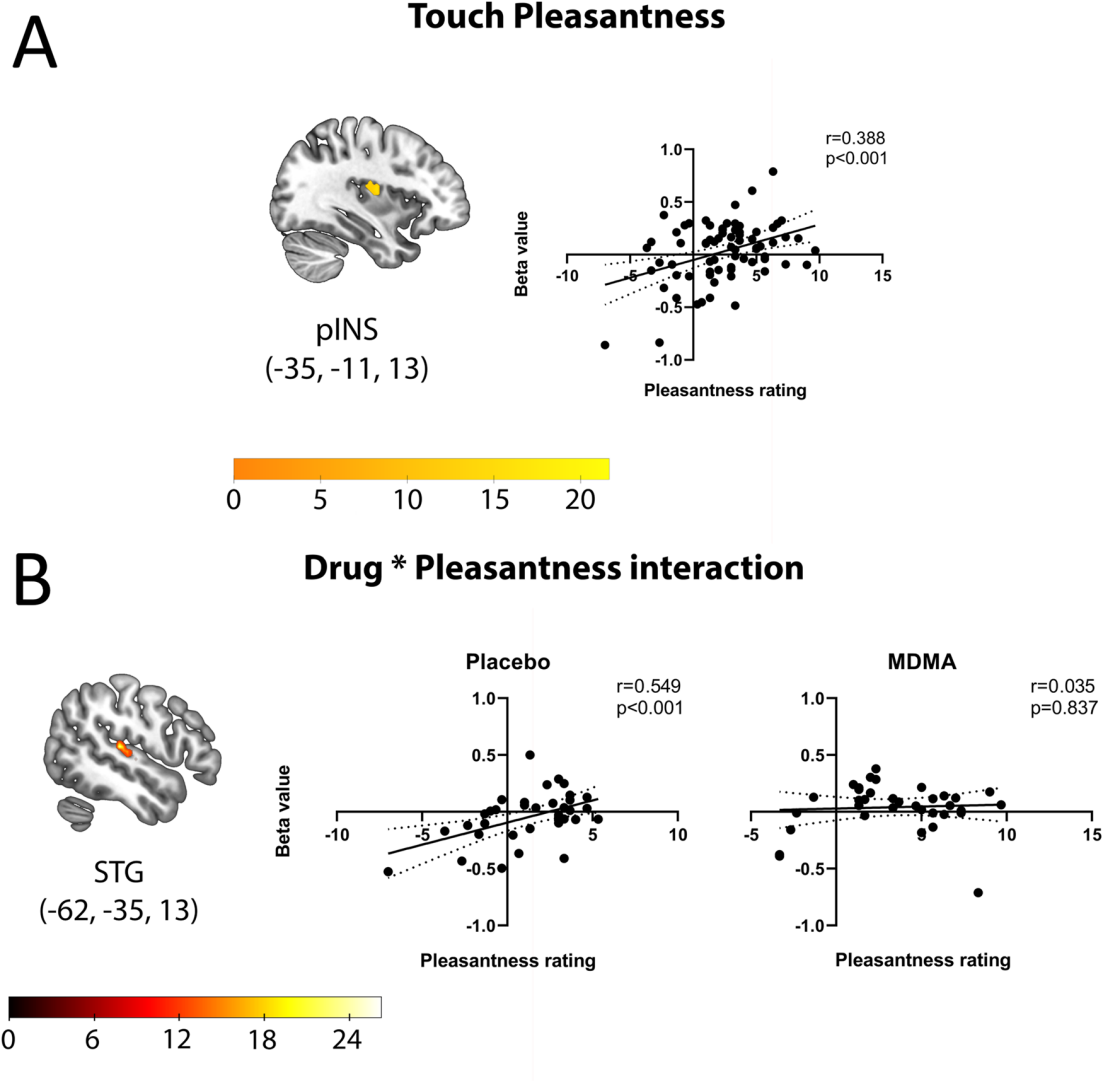
Relationships between pleasantness rating covariate and BOLD activation. (A) Posterior insula (PI) responses ipsilateral to the touch stimulation showed a positive relationship with subjective pleasantness ratings, regardless of drug or stroking velocity, with higher BOLD in individuals who experienced touch as more pleasant. (B) BOLD activation in superior temporal gyrus (STG) showed an interaction between drug and pleasantness regardless of drug or stroking velocity, with a positive relationship during the placebo session and a less positive relationship during the MDMA session. This pattern was also observed in clusters in left lingual gyrus, right supplementary motor area, left paracentral lobule, and the left calcarine gyrus (not depicted). Scatterplots represent each participant average pleasantness value in each of the four conditions. MNI coordinates for peak voxels in parentheses; images shown in radiological convention (left side of image is right side of brain). Color bars represent*F*statistic values.

Ratings on pleasantness of touch (regardless of whether it was slow or fast) were positively correlated with the BOLD signal in three clusters after controlling other variables: right and left supplementary motor area, and in the left posterior insula (Fig. 3A).

### 3.9 Interaction between drug condition and pleasantness ratings on BOLD activation

The pleasantness rating covariate interacted with the drug treatment factor in seven clusters. In each of these clusters, BOLD activation covaried more positively with pleasantness under placebo than under MDMA. These were the left lingual gyrus, the right supplementary motor area, the left superior temporal gyrus (two clusters), the left paracentral lobule, and the left calcarine gyrus (two clusters,Fig. 3B).

## 4 Discussion

As predicted, the perceived pleasantness of touch was greater for a 1.5 mg/kg dose of MDMA than for a lactose placebo. Although the slower stroking speed was perceived as more pleasant than the faster one, both speeds showed a general increase for MDMA compared with placebo, in contrast to previous findings (Bershad et al., 2019). BOLD responses to touch modulated activation in primary sensory cortices for MDMA relative to placebo, regardless of stroking speed. MDMA also modulated cortical responses to touch in sensorimotor, occipitoparietal, and medial prefrontal cortices. BOLD changes in sensorimotor and occipitoparietal clusters also covaried with changes in endogenous OT levels, which were greater during the MDMA session than placebo. Posterior insula activation increased with pleasantness ratings but did not show a preference for the slower stroking speed.

### 4.1 Effects of MDMA on affective touch

Perceived pleasantness and desirability (“wanting”) of both slow and fast touch were greater for MDMA than placebo (Fig. 1). Participants rated the slower stroking speed (3 cm/s) as being more pleasant than the faster speed (30 cm/s), which is consistent with previous results (Crucianelli et al., 2022;Mayo et al., 2018;Novembre et al., 2021;Perini et al., 2021;Sailer et al., 2020). Both stroking speeds were rated as more intense during the drug session (Fig. 1), suggesting that the drug effect was not limited to subjective, affective, or motivational qualities. Further research will be needed to investigate the selectivity of MDMA for subjective responses in the affective domain. The time course and magnitude of the subjective and cardiovascular effects of MDMA were similar between the present study and a previous behavioral study on affective touch (Bershad et al., 2019) (Fig. 1).

In the current study, we used a placebo control rather than incorporating an active control like*d-*amphetamine. Although an active control can minimize functional unblinding effects with subjective responses, using an inactive placebo was essential for comparing brain activation with “typical” responses to touch. Stimulants such as*d*-amphetamine also alter neurotransmission and brain activation patterns, making a placebo control necessary as a first step to assess response differences. Active controls will be useful to ascertain MDMA-specific response differences, over and above any common amphetamine-related responses, in future research.

The underlying molecular mechanisms that contribute to MDMA’s effects on affective touch are still unknown, though serotonergic signaling has been heavily implicated. Rodent studies suggest that involvement of serotonergic 5-HT1A receptors may mediate affiliative behaviors (Morley et al., 2005;Thompson et al., 2007). In humans, subjective- and mood-enhancing effects of MDMA are mediated by 5-HT2 (serotonin) and D2 (dopamine) receptor binding (Liechti & Vollenweider, 2001;van Wel et al., 2012), but the extent to which these receptors contribute to enhanced response to affective touch is not currently known, and thus requires examination in future studies.

### 4.2 MDMA effects on BOLD activation

BOLD responses to touch in primary somatosensory and motor regions surrounding the central sulcus (SI/MI;Fig. 2;Table 3) were modulated by drug, showing a main effect in which BOLD for MDMA was greater than for placebo. These findings support the hypothesis that MDMA affects tactile cortical pathways at a relatively early processing level. It also implies that it modulates both sensory and motor processing, not limited to primary cortices but also cerebellum ipsilateral to the response hand. The effect sizes in these sensorimotor and cerebellar clusters were among the strongest in the study. The activation of sensorimotor cortices by MDMA by passive touch stimulation suggests that MDMA may also affect readiness to engage in active touch, especially in a nonlaboratory setting, though this requires further experimentation.

The proposition that MDMA alters affective and contextual processing independently of sensory feature processing was not strongly supported. MDMA did not selectively modulate BOLD activation in temporal and parietal regions previously implicated in the integrative processing of touch stimulation. The absence of activation in parietal and temporal regions for drug vs. placebo may be related to procedural constraints on ecological social interaction, such as being stroked with a soft brush by an experimenter in the MR environment. However, MDMA modulated the ventromedial prefrontal cortex (mPFC) bordering on orbitofrontal cortex (OFC;Fig. 2), a key region strongly implicated in situations requiring attention, emotion, social processing, flexible behavior, the generation of appropriate social responses, and response inhibition (e.g., (Franklin et al., 2017)).

MDMA also modulated BOLD responses in primary visual cortex in and around the calcarine sulcus. The effect sizes in these clusters were also among the most robust in the study. The moving tactile stimulus may have evoked visual imagery, as it implies the presence of a nonvisible person delivering the touch. The content of visual imagery can be decoded from deeper layers of primary visual regions V1 (Bergmann et al., 2024). MDMA may facilitate such imagery, as suggested by previous research indicating altered serotonergic signaling in occipital cortex, and thus altered visual perception (White et al., 2013). The preferential activation in middle and superior occipital gyri during the placebo session may reflect such alteration or capture contrastive fluctuations, but we report these findings in the absence of a hypothesis or strong interpretation.

It remains to be investigated whether MDMA affects processing in these pathways across various stimuli, social contexts, and motivational variables. This includes different features of touch stimulation (e.g., texture, temperature), interactions with familiar vs. unfamiliar social partners, or active rather than passive touch (e.g., motivated action). Given that real-world social interactions are complex and challenging to capture in a controlled experimental setting, future studies examining the effects of MDMA should take these factors into consideration.

### 4.3 Interactions among drug, ΔOT, and BOLD

Consistent with previous findings (Holze et al., 2020;Kirkpatrick et al., 2014), changes in plasma OT were greater after MDMA administration than after placebo (Fig. 1C). A relationship between MDMA and central OT release may be mediated by serotonergic signaling, via 5-HT1A receptor activation in the hypothalamus (Atila et al., 2023;Forsling et al., 2002). Although it has been postulated that the prosocial behavioral effects of MDMA involve its effects on OT, we saw no correlation between drug-induced change in OT and ratings of touch pleasantness (for either faster or slower speeds) (Dumont et al., 2009;Handlin et al., 2023;Kamilar-Britt & Bedi, 2015).

MDMA administration consistently increases plasma OT (Francis et al., 2016;Holze et al., 2020;Kirkpatrick et al., 2014). Although saliva OT has also shown changes with similar doses of MDMA as in the present study (Molla et al., 2023), we chose to collect blood plasma samples in this study for both methodological and practical reasons. First, in our previous unpublished tests, salivary OT values have been inconsistent, often falling below a detectable threshold for baseline and placebo sessions, making it difficult to determine the true magnitude of change. Additionally, dry mouth is a common side effect of MDMA, posing potential challenges to saliva collection.

**Fig. 4.**
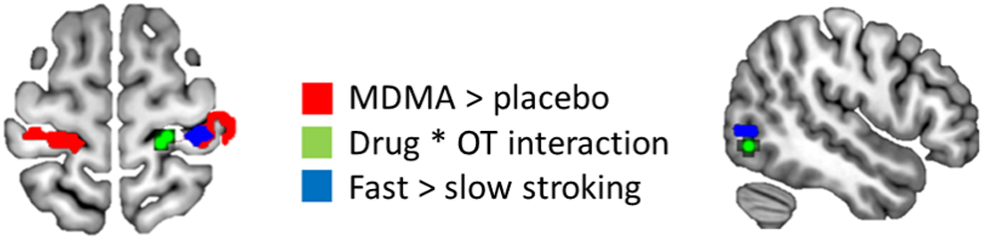
Adjacent or overlapping BOLD activation in sensorimotor (left-hand image) and occipitotemporal (right-hand image) cortices for MDMA vs. placebo (red), interaction between the drug factor and change in plasma OT levels (green), and fast vs. slow touch (blue). Images shown in radiological convention (left side of image is right side of brain). Clusters color coded for corresponding contrast in center of figure.

On the cortical level, MDMA-related changes in BOLD activation covaried with OT changes in a cluster within the SI/MI activations showing a main effect of drug (Table 2,Fig. 2). BOLD–OT changes also showed an interaction with drug in an occipitotemporal region encompassing motion-selective area MT+ (Fig. 4), which is mainly associated with detection of moving visual stimuli as well as detection of tactile motion (Beauchamp et al., 2008;Hagen et al., 2002); though seeJiang et al. (2015). Activation in this region was recently observed for reciprocal touch-based coordination of hand movements between two people (Chauvigne et al., 2019), suggesting a contribution to tactile-mediated social interaction, which aligns with MDMA’s reported role in affective social behavior.

The interaction in this MT+ cluster was likely driven by a negative relationship between OT change and BOLD during the MDMA session, in which individuals showing greater OT change also showed lesser BOLD change in this cluster. However, during the placebo session, changes in OT levels were not correlated with BOLD activation here. This suggests that MDMA modifies a BOLD–OT relationship in this region if responses during the placebo session are taken as typical.

The MT+ cluster was directly adjacent to a cluster that showed increased BOLD responses for faster stroking (Fig. 4, right). This cluster extended into the posterior superior temporal sulcus (pSTS) and nearby temporoparietal regions that have been implicated in affective touch (Gordon et al., 2013;Kaiser et al., 2016) as well as in multisensory integration and aspects of inferring the mental states of others (Allison et al., 2000;Braunsdorf et al., 2021;Igelstrom & Graziano, 2017). pSTS has indirect connections with other regions that play strong roles in reward learning and social affiliation (Depue & Morrone-Strupinsky, 2005) and neuromodulation by OT (Gothard & Fuglevand, 2022;Mosher et al., 2016;Xiao et al., 2018). These findings in MT+ and STS are currently more suggestive than conclusive, given the present sample and effect sizes, but will be further explored in future studies.

### 4.4 Relationship between pleasantness and BOLD

The reported pleasantness of touch, regardless of speed or drug condition, was positively correlated with BOLD activation in bilateral supplementary motor area (SMA) and ipsilateral posterior insular cortex (PI). SMA is a premotor region on the medial surface of the cortex associated with, among other functions, action preparation and execution (Mueller et al., 2007). PI and nearby parietal operculum (PO) are widely implicated in affective touch (Morrison, 2016;Morrison et al., 2011;Perini et al., 2015). Patients with PI/PO lesions resulting from stroke reported reduced touch intensity and pleasantness compared with controls (Kirsch et al., 2020). However, evidence for a relationship between subjective pleasantness and PI activation is mixed, with some studies reporting a correlation (Kress et al, 2016) and others reporting no correlation (Morrison et al., 2011). The covariance between BOLD and pleasantness in PI ipsilateral to stimulation and contralateral to the response hand (for posttrial ratings) may indicate a contribution of task in the context of the experimental paradigm, but the extent to which task-related factors influence affective touch processing remains underexplored.

Whereas the PI was related to pleasantness of touch regardless of speed (Fig. 3A), BOLD responses here did not selectively increase for the slower speed. This was consistent with a previous study using an identical experimental paradigm (Perini et al., 2021), another using a different paradigm (Case et al., 2016), but not others using similar methods (Morrison et al., 2011;Perini et al., 2015). This suggests that design factors affect the speed selectivity in this region. For example, differences between block and event-related designs, task and attentional demands, and timing tradeoffs between the duration and frequency of arm stroking may influence relative activation effects (Morrison et al., 2011). Here, for example, a greater number of strokes were delivered for the faster than for the slower speed during the blocked 12s trials. The MT+ region showed increased activation for fast (higher frequency) vs. slow (lower frequency) touch, consistent with its putative role in tactile motion detection (Beauchamp et al., 2008;Hagen et al., 2002;Jiang et al., 2015).

Comparing the faster and slower stroking speeds can capture neural systems associated with affective touch (Löken et al., 2009), in particular activation of a specific subtype of afferent nerve fiber in humans, the C-tactile or CT afferent. CT afferent activation correlates on a group level with ratings of touch pleasantness across different stroking speeds (Löken et al., 2009). The slower speed is also less likely than faster stroking to evoke “frowning” activation of the*corrugator supercilii*muscles in the forehead (Mayo et al., 2018), suggesting that it attenuates negative affective facial responses to touch.

During the placebo session, individuals who found the touch stimulation more pleasant also showed greater BOLD increases in superior temporal gyrus (STG), previously implicated in touch pleasantness (Davidovic et al., 2016) (Fig. 3B). However, this relationship was not present during the MDMA session. This suggests that MDMA may affect touch pleasantness nonselectively, rather than selectively enhancing touch stimuli preferred under placebo.

While future studies with larger sample sizes are still necessary, the current findings indicate that MDMA enhances the affective perception of touch in addition to altering cortical processing of touch in primary sensorimotor regions, visual cortices, and cerebellum. They also provide preliminary evidence that OT modulates this effect, suggesting that the hormone may affect the integration of touch information in brain-wide networks. Touch is also a key component in current treatments under development for PTSD (Mithoefer, 2017). According to the Multidisciplinary Association for Psychedelic Studies (MAPS) treatment manual, touch is “an important catalyst to healing during both the MDMA-assisted sessions and the follow-up therapy.” Improving our understanding of the effects of MDMA on affective touch perception may thus be a key component for exploring the effectiveness of this new treatment modality (Luoma et al., 2023).

## Data Availability

The code used in this experiment and the data are available upon request.
